# Antibiotic Prophylaxis of Infective Endocarditis in Dentistry: An Update on the ESC, AHA, and NICE Guidelines

**DOI:** 10.3390/jcm15197434

**Published:** 2026-09-24

**Authors:** Julia Bijoch

**Affiliations:** Collegium Medicum–Faculty of Medicine, WSB University, 41-300 Dabrowa Gornicza, Poland; julia.bijoch@wsb.edu.pl

**Keywords:** infective endocarditis, antibiotic prophylaxis, viridans group streptococci, bacteraemia, dental procedures, clinical guidelines, antimicrobial resistance, oral health

## Abstract

Infective endocarditis (IE) is a rare but life-threatening infection of the endocardial surface of the heart, usually arising when circulating microorganisms colonise the cardiac valves; it carries an in-hospital mortality of roughly 15–30%. Because oral viridans group streptococci cause a substantial proportion of community-acquired cases, antibiotic prophylaxis (AP) before invasive dental procedures has historically been central to prevention, placing dental practitioners at the centre of a long-standing and unsettled debate. This narrative review synthesises the contemporary evidence and the divergence between major guideline bodies. The 2023 European Society of Cardiology (ESC) guidelines raised AP for high-risk patients to a Class I recommendation, and the 2021 American Heart Association (AHA) statement remains closely aligned, restricting AP to those at highest risk of adverse outcome. The United Kingdom’s National Institute for Health and Care Excellence (NICE), by contrast, has advised against AP since 2008 (and, since a 2016 amendment, against its routine use). This divergence has produced an international discordance that leaves clinicians and patients facing conflicting advice. This review examines the pathogenesis and microbiology of oral-origin IE, the conditions and procedures that define risk, and the contested epidemiological evidence, including population-level data linking reduced prescribing to rising IE incidence. It then discusses the implications for clinical practice and the central role of oral health. The evidence base remains observational and associative rather than causal; definitive randomised data are lacking. The review argues that, pending stronger evidence, dentists should identify the framework governing their jurisdiction, apply it consistently, prioritise oral health, and share documented decisions with patients.

## 1. Introduction

Infective endocarditis (IE) is a microbial infection of the endocardial surface of the heart, most often involving the cardiac valves. Although its annual incidence is low, of the order of 3 to 10 cases per 100,000 person-years, it remains one of the most serious infections encountered in medicine, with in-hospital mortality commonly reported between 15% and 30% and substantial one-year mortality thereafter [1,2]. Its incidence has been rising in high-income countries, a trend attributed in large part to the ageing of the population and to the growing use of prosthetic and transcatheter valve implants, which expand the pool of patients carrying the cardiac risk factors most strongly associated with IE [3]. The disease arises when circulating microorganisms adhere to a region of damaged or abnormal endocardium and establish an infected vegetation; the oral cavity is one of the principal portals through which the responsible organisms, particularly viridans group streptococci, enter the bloodstream [4].

This biological link between the mouth and the heart places dentistry at the centre of IE prevention. For decades, the dominant preventive strategy was the administration of antibiotic prophylaxis (AP) before dental procedures expected to provoke bacteraemia, on the rationale that suppressing transient bloodstream seeding might prevent valvular infection in susceptible individuals. Over the past two decades, however, that rationale has been progressively re-examined. Successive guideline revisions have narrowed the indications for prophylaxis, reflecting growing doubt about its efficacy, concern about adverse drug reactions and antimicrobial resistance, and recognition that everyday activities such as chewing and toothbrushing produce cumulative bacteraemia far exceeding that of a single dental visit.

As a result, the major guideline bodies no longer agree. The 2023 European Society of Cardiology (ESC) guidelines and the 2021 American Heart Association (AHA) scientific statement converge on a restricted, risk-stratified use of prophylaxis for patients at the highest risk of an adverse outcome [3,4]. The United Kingdom’s National Institute for Health and Care Excellence (NICE), by contrast, has advised against prophylaxis since 2008; since 2016, its guidance has stated that prophylaxis is not recommended routinely. NICE has reaffirmed this position in substance at each subsequent review, most recently in 2024, and it now diverges from international practice [5,6]. For the dental practitioner, this discordance has direct practical consequences: it determines whether an individual patient should receive an antibiotic, and it has clinical, ethical, and medicolegal implications.

This narrative review provides an update oriented to the dental reader. It summarises the pathogenesis, microbiology, and risk determinants of oral-origin IE; sets out and compares the current ESC, AHA, and NICE positions, with the ESC/AHA-versus-NICE divergence as its central axis; appraises the contested epidemiological evidence on which the debate turns; and considers the practical implications for clinical dentistry, including the importance of shared, well-documented decision-making where guidance conflicts. Throughout, a deliberate distinction is drawn between what the evidence demonstrates, what it suggests, and what remains unresolved.

## 2. Methods

This is a narrative review. PubMed/MEDLINE, EMBASE, Scopus, Web of Science, and the Cochrane Library were searched from January 2000 to June 2026, combining terms for infective endocarditis, antibiotic prophylaxis, dental or oral procedures, bacteraemia, viridans streptococci, and the named guideline bodies (European Society of Cardiology, American Heart Association, National Institute for Health and Care Excellence). The websites of the ESC, AHA, NICE, the American Dental Association (ADA), and the Scottish Dental Clinical Effectiveness Programme (SDCEP) were consulted directly for primary guidance documents. Reference lists of key articles and guidelines were screened for additional sources. Priority was given to primary guideline documents, large epidemiological studies, systematic reviews, and meta-analyses. Selected earlier landmark studies published before 2000 were also included where directly relevant. Given the narrative design, no formal protocol was registered and no quantitative synthesis was applied, and the selection of sources necessarily reflected the author’s judgement of relevance; this is acknowledged as a limitation.

MeSH terms were exploded in PubMed/MEDLINE (Endocarditis, Bacterial; Antibiotic Prophylaxis; Dental Care); the other databases were searched using free-text keywords. The final search was executed in June 2026.

Eligibility criteria were applied as follows. Included were: primary guideline and surveillance documents from the ESC, AHA, NICE, ADA and SDCEP; systematic reviews and meta-analyses; population-level, cohort, case–control, case-crossover and interrupted time-series studies reporting IE incidence or prophylaxis exposure; and mechanistic studies of oral bacteraemia. Excluded were: case reports and small case series; conference abstracts without full text; narrative commentaries without new data or new analysis, except where cited to represent a stated professional position; studies confined to non-oral portals of entry, prosthetic-device infection unrelated to dental care, or paediatric populations reported without extractable IE outcomes; and articles in languages other than English. Records were screened by title and abstract and then at full text by the sole author. Where studies were discordant, all were retained and reported, and the discordance is discussed explicitly in Section 7 rather than resolved by selection.

Screening and selection were performed by the sole author, so no inter-rater reliability could be calculated and record counts were not maintained prospectively; no PRISMA flow diagram is therefore presented; the search strategy is reproducible, but the screening process cannot be independently audited. To mitigate this, every guideline position reported here was extracted from the primary document rather than a secondary summary and verified against the source text and its recommendation class, and the pivotal observational studies were appraised narratively against the ROBINS-I domains [7], with the judgements reported in Section 7. No numerical quality score was assigned and no pooling attempted, consistent with the narrative design.

## 3. Pathogenesis and Microbiology of Oral-Origin Infective Endocarditis

The classical model of IE pathogenesis begins with endothelial injury or abnormal flow across a structurally altered valve, which promotes deposition of platelets and fibrin and the formation of a sterile thrombotic lesion termed nonbacterial thrombotic endocarditis. During an episode of bacteraemia, circulating organisms adhere to this lesion, proliferate, and become encased within a protective biofilm of platelets and fibrin, forming the mature infected vegetation. The relative inaccessibility of organisms within this matrix to both host defences and antibiotics underlies the difficulty of treating established disease and provides the conceptual basis for attempting prevention at the point of bacteraemia.

Viridans group streptococci, commensal inhabitants of the oral cavity and dental plaque, are among the organisms best adapted to this sequence. Their surface adhesins bind avidly to platelet and fibrin components, and they are implicated in a considerable share of community-acquired native-valve IE, historically estimated at around a third to a half of such cases, although staphylococci have become increasingly prominent overall, particularly in healthcare-associated and prosthetic-material disease [1,2]. It is the oral streptococcal fraction that is theoretically amenable to dental prophylaxis; IE caused by staphylococci, enterococci, or other organisms is largely unrelated to dental care and would not be expected to respond to it. This distinction is well illustrated by endocarditis complicating prosthetic and transcatheter valves, in which staphylococci and enterococci predominate and carry a high mortality, and in which enterococci in particular are often inadequately covered by standard periprocedural prophylaxis, a burden of disease that lies outside the scope of any dental intervention [8]. Endocarditis arising after transcatheter aortic valve implantation is moreover frequently difficult to recognise, as a substantial proportion of cases lack the classic echocardiographic signs, which can delay diagnosis and worsen outcomes. This has a direct practical implication for the dental team, which concerns diagnostic vigilance rather than prophylaxis. If a patient with a transcatheter or prosthetic valve develops unexplained fever, malaise, or other constitutional symptoms in the weeks after an invasive dental procedure, a high index of suspicion for IE should be maintained and prompt referral for blood cultures and specialist assessment arranged, even where an initial echocardiogram is unremarkable or equivocal, since a normal early study does not exclude the diagnosis in this group [9]. This applies regardless of whether the dental procedure had any causal role—which, given the microbiology set out above, it frequently will not have had—because the dentist may be the first clinician to whom such a patient reports, and because prophylaxis, where it was given, does not exclude the diagnosis either.

A central and frequently underappreciated point concerns the source and magnitude of oral bacteraemia. Invasive dental procedures such as extraction do produce measurable bacteraemia, but so do routine daily activities: chewing, toothbrushing, flossing, and other ordinary oral functions. Because these activities occur many times every day throughout life, their cumulative contribution to bloodstream exposure has been estimated to exceed, by several orders of magnitude, the transient bacteraemia attributable to occasional dental procedures [10]. This observation has two consequences that run through the entire prophylaxis debate: it casts doubt on whether targeting dental procedures alone can meaningfully reduce IE risk, and it elevates the maintenance of good oral health, which reduces the bacterial load available for everyday seeding, to a position of primary importance.

## 4. Risk Stratification: Cardiac Conditions and Dental Procedures

Contemporary guidance frames risk along two axes: the patient’s underlying cardiac condition and the nature of the dental procedure. The decisive conceptual shift embodied in modern guidelines is that prophylaxis, where recommended at all, is reserved not for everyone with a theoretically increased lifetime risk of acquiring IE, but specifically for those in whom an episode of IE would be most likely to result in a catastrophic outcome: death or severe morbidity. This reframing, from cumulative acquisition risk to adverse-outcome risk, substantially reduced the population considered eligible; the case–control evidence that dental procedures were not associated with an increased risk of IE was among the findings that prompted it [11]. The criteria are grouped here so that the points of inter-guideline divergence become visible, rather than in the order any guideline presents them. The categories are consensus groupings rather than empirically derived strata. They rest on observed case-fatality and morbidity following IE in each condition, on pathophysiological reasoning, and on case–control and registry data, but no study has compared prophylaxis against no prophylaxis within each category, and the thresholds separating highest-risk from moderate-risk conditions were set by expert judgement rather than estimated. The boundaries between categories are therefore less clearly defined than their tabular presentation suggests, a point that matters because it is precisely at those boundaries that the ESC and AHA diverge.

Across the ESC and AHA frameworks, the cardiac conditions placed in the highest-risk category are broadly consistent and include: a prosthetic cardiac valve or prosthetic material used for valve repair; a previous episode of infective endocarditis; and specified forms of congenital heart disease (CHD), notably unrepaired cyanotic congenital heart disease, and defects repaired with prosthetic material during the first six months after the procedure, or indefinitely where a residual defect persists adjacent to the prosthetic material. The AHA additionally includes cardiac transplant recipients who develop valvulopathy in the highest-risk group, whereas the ESC treats heart transplant recipients as a Class IIb consideration [3,4]. These conditions are summarised, with the points of inter-guideline difference, in Table 1.

On the procedural axis, prophylaxis, when indicated by cardiac status, is directed at dental procedures involving manipulation of the gingival tissue or the periapical region of the teeth, or perforation of the oral mucosa. Routine, non-invasive interventions such as superficial restorations above the gingival margin, radiographs, the placement or adjustment of removable prosthodontic or orthodontic appliances, and the shedding of deciduous teeth are not considered to warrant prophylaxis. The unifying principle across all current frameworks, including NICE, is that meticulous oral health and regular dental care matter more than any single antibiotic dose. The bacteraemia data discussed in Section 3 require careful interpretation in this context. That invasive procedures produce more endocarditis-related bacteraemia than ordinary oral function is supported by randomised data: cumulative incidence across six blood draws was 23% after toothbrushing, 60% after extraction with placebo, and 33% after extraction with amoxicillin [10]. The per-event gradient is real; what Section 3 establishes is that lifetime cumulative exposure is nonetheless dominated by toothbrushing, because it recurs daily and extraction does not. The two findings are complementary. The gradient justifies treating extraction differently from toothbrushing, but no study grades IE risk, as opposed to bacteraemia, across the procedures on either side of the boundary the guidelines set; the classification is best read as a defensible operational convention rather than a validated risk boundary.

### Periodontal Care and Lifelong Maintenance in the At-Risk Patient

Periodontal treatment is a course of care extending over months and, in maintenance, over a lifetime; it therefore raises questions the guidelines answer only obliquely.

The first question is which periodontal procedures fall within the definition. The ESC and AHA trigger—manipulation of gingival tissue or the periapical region—captures subgingival scaling, root planing and periodontal surgery, and also, less obviously, periodontal probing and supragingival scaling, since both can provoke bleeding and measurable bacteraemia with IE-related organisms. Under ESC and AHA guidance, prophylaxis is normally recommended for these procedures in high-risk patients, as they were in the United Kingdom before 2008. The empirical picture is more complex than the classification implies: in a cohort study of nearly eight million individuals, the association with IE in high-risk patients was concentrated in extractions (OR 9.22; 95% CI 5.54–15.88) and oral surgery (OR 20.18; 95% CI 11.22–36.74), whereas scaling was associated with a lower incidence of IE (OR 0.46; 95% CI 0.26–0.81), most plausibly because patients who attend for regular scaling have better oral health. The parallel case-crossover analysis, in which each patient serves as their own control and time-invariant characteristics such as oral hygiene are thereby held constant, showed the same pattern (extractions OR 11.08, 95% CI 7.34–16.74; oral surgery OR 50.77, 95% CI 20.79–123.98; scaling OR 0.69, 95% CI 0.47–1.02, not significant) [12]. This reflects the healthy-adherer effect discussed in Section 7 and illustrates why the invasive/non-invasive dichotomy, although operationally useful, does not correspond precisely to risk.

The second concerns repeated exposure. Quadrant-by-quadrant debridement may span several appointments within weeks, and maintenance implies recall every three to four months indefinitely; covering every visit would convert a single-dose intervention into recurrent antibiotic exposure over decades—a pattern that antimicrobial stewardship principles (Section 8) seek to avoid. Where repeated prophylaxis is unavoidable, the AHA suggests an interval of 10 days between procedures requiring prophylaxis [4]; consolidating treatment into fewer, longer visits further reduces both cumulative exposure and selection pressure.

The third, and the most consequential in practice, concerns sequencing. Periodontal inflammation should not be regarded merely as an indication for prophylaxis before treatment; it is itself the dominant source of the everyday bacteraemia described in Section 3, and the rationale for treating it is therefore stronger than that for providing antibiotic cover during treatment. The logic of the bacteraemia argument implies that a high-risk patient with untreated periodontitis is exposed to more oral streptococcal seeding between appointments than the appointments themselves contribute, and that leaving disease untreated out of caution about prophylaxis reverses the balance of risk. Where a patient is awaiting valve surgery, this becomes a matter of timing: dental assessment and the elimination of active infection ideally precede elective implantation, since the postoperative period is when the consequences of an oral-origin bacteraemia are gravest.

## 5. The Evolution of Prophylaxis Guidelines

Understanding the present disagreement requires a brief historical orientation. The following overview is deliberately selective and focuses on the aspects that explain the present divergence: the progressive narrowing of the indication and the differing responses of each body to the same absence of trial evidence. For much of the late twentieth century, expansive antibiotic prophylaxis was recommended for a wide range of cardiac conditions before numerous dental and non-dental procedures, on a largely empirical and precautionary basis. The early twenty-first century brought a decisive reappraisal, driven by three converging concerns: the absence of randomised evidence demonstrating that prophylaxis actually prevents IE; mounting awareness of the harms of antibiotics, including anaphylaxis and the societal cost of antimicrobial resistance; and the recognition, discussed above, that cumulative everyday bacteraemia overshadows procedure-related exposure [13].

In 2007, the AHA substantially narrowed its recommendations, restricting prophylaxis to the highest-risk cardiac categories and abandoning prophylaxis based on lifetime acquisition risk alone; the American Dental Association endorsed this approach as it related to dentistry [14,15]. The 2021 AHA scientific statement reviewed the intervening evidence and recommended no substantive change, reaffirming the restricted high-risk approach while emphasising oral health; it also removed clindamycin from the recommended regimens because of its disproportionate risk of adverse effects, including *Clostridioides difficile* infection [4]. The 2023 ESC guidelines, having reviewed the accumulated evidence, retained the high-risk focus but m moved in the opposite direction to NICE, strengthening the recommendation for prophylaxis in high-risk patients to Class I and allowing that it may be considered on an individual basis in moderate-risk patients (Class IIb) [3].

NICE reached the opposite conclusion from the same evidence base because it applied a higher evidentiary threshold and treated the intervention, rather than its omission, as the option requiring positive justification. Its 2008 guideline concluded that the evidence did not justify routine prophylaxis and advised against it for all patients, a position more categorical than that adopted by other bodies, and one that follows from its stated requirement for demonstrated efficacy rather than from any disagreement about the underlying data [5]. A 2016 amendment softened the wording, inserting the word “routinely” so that the guidance read that prophylaxis is not recommended routinely, but it did not restore a positive recommendation [6]. This amendment followed NICE’s 2015 review of the evidence [5] and was a change in wording rather than of substance; the underlying recommendation has not been materially revised since. In October 2024, NICE undertook an exceptional surveillance review of the evidence, including the contemporary meta-analytic data; it concluded that robust evidence remained lacking and left the substance of the CG64 recommendation unchanged. The same review now signposts the implementation advice of the Scottish Dental Clinical Effectiveness Programme (SDCEP), which helps identify the subset of higher-risk patients warranting special consideration for prophylaxis [16]. The core NICE position (no positive recommendation for routine prophylaxis) has thus remained essentially unchanged, even as its practical framing has been nuanced. This trajectory left United Kingdom guidance increasingly divergent from international practice as the ESC and AHA reaffirmed and, in the ESC’s case, strengthened a role for prophylaxis in the highest-risk group.

## 6. The Central Divergence: ESC/AHA Versus NICE

The core of the current controversy may be summarised as follows. The ESC and AHA, while differing in emphasis and in a few specific criteria, agree that a defined group of highest-risk cardiac patients should receive antibiotic prophylaxis before invasive dental procedures. NICE, alone among major bodies, advises against routine prophylaxis for any group. A dental patient with a prosthetic heart valve facing an extraction would, therefore, in much of Europe and North America, be offered prophylaxis as a matter of course, yet, under a literal reading of NICE guidance in England and Wales, might receive none. This discordance is a source of uncertainty in clinical practice.

It is important to characterise the ESC and AHA as broadly concordant rather than identical. Both restrict prophylaxis to the highest-risk categories and both prioritise oral health. The differences are at the margins: the ESC explicitly contemplates individualised prophylaxis in moderate-risk patients (Class IIb), an option not included in the AHA statement; the AHA places cardiac transplant recipients with valvulopathy in the highest-risk group, whereas the ESC treats heart transplant recipients as a Class IIb consideration—a move towards the AHA, since the 2015 ESC guidelines had explicitly declined to recommend prophylaxis for this group [13]; and the two differ in minor respects regarding certain repaired congenital lesions and conduits. For the dental practitioner, these distinctions rarely change management of the clearly highest-risk patient, but they matter at the boundaries and they illustrate that even “aligned” guidelines are not interchangeable.

The NICE position should be understood on its own terms rather than dismissed as incorrect. It rests on a defensible reading that the efficacy of prophylaxis has never been demonstrated in a randomised trial, that the number of cases potentially preventable is very small even under optimistic assumptions, and that the harms and costs of widespread antibiotic use are real. Its critics counter that absence of randomised evidence is not evidence of absence of effect, that the consequences of an IE case are catastrophic and the harm of a single prophylactic dose minimal, and that observational data have since shifted in favour of a protective effect [17,18]. The NICE position may be summarised in its strongest form as follows. Even if the pooled relative risk of approximately 0.41 [18] is taken at face value, prophylaxis before invasive dental procedures could avert only a small minority of IE cases in high-risk patients, since such procedures account for only a fraction of cases; the number needed to treat is correspondingly very large, because the absolute risk after any single procedure is very low, even in a high-risk patient. Against that, every dose contributes to selection pressure at population level. The disagreement is therefore not a simple matter of one body being right and another wrong; it is a genuine difference in how the same imperfect evidence is weighed against the same competing harms. All three bodies accept that no randomised evidence exists, that the observational signal is real but confounded, and that the number of preventable cases is small; what separates them is the evidentiary threshold each applies before recommending an intervention. That threshold is not itself derivable from the data, which is why the contradiction cannot be settled by another observational study.

Across most of continental Europe, this divergence does not arise in the same way, because the ESC guidelines function as the operative standard and are disseminated through national cardiological societies, which typically translate and endorse them rather than issue competing advice [3]. The practical implication is that a dentist working anywhere within the ESC-aligned European setting will generally follow the same risk-stratified approach centred on the highest-risk group, and the disagreement described above is therefore felt most acutely in the United Kingdom rather than across Europe as a whole.

## 7. The Contested Epidemiological Evidence

Because no randomised controlled trial has ever tested whether antibiotic prophylaxis prevents IE, and because the rarity of the disease makes such a trial very difficult to power adequately—while withholding prophylaxis from an identified highest-risk patient raises an ethical objection that individual randomisation cannot easily answer—the debate has so far relied on observational and population-level evidence. This evidence is inconsistent, and a balanced appraisal requires consideration of studies on both sides.

The most influential study supporting a protective role examined IE incidence in England before and after the 2008 NICE guidance [17]. It reported that prescriptions of prophylaxis fell substantially after the guideline, and that the incidence of IE rose significantly above the projected historical trend, with a meaningful number of additional cases per month by the end of the study period; notably, the increase was observed across both higher-risk and lower-risk groups. The authors were careful to state that their interrupted time-series design could not establish causation, only temporal association, and that other secular trends in the epidemiology of IE could contribute. Nonetheless, the temporal coincidence of falling prophylaxis and rising incidence has been widely cited as evidence that withdrawing prophylaxis may have caused harm. This inference is not supported by the study design. The study’s own finding that incidence rose in both higher-risk and lower-risk groups is hard to reconcile with a causal effect of withdrawing prophylaxis, since prophylaxis was never indicated in the lower-risk group. A more parsimonious interpretation is that a common cause—an ageing population, more prosthetic and transcatheter implantation, rising injection drug use, improved ascertainment and coding—drove incidence upward in both groups, and that the guideline change is confounded with calendar time. An interrupted time-series can identify an effect only if the counterfactual trend is correctly specified; here, the pre-intervention trend was itself rising, and the estimate is sensitive to the functional form chosen for extrapolation. This is ecological inference: an association between two population-level time series cannot license a claim about individual-level causation.

Other studies have not consistently supported this finding. An earlier before-and-after analysis, by an overlapping group of investigators, had found no statistically significant increase in IE cases following the same guideline, although it was able to exclude only increases above a certain threshold [19]. Studies from other settings have likewise been inconsistent: a Swedish national cohort study in high-risk individuals could not confirm that invasive dental procedures were more frequent before IE than expected, including during the period when Swedish dentistry had ceased prophylaxis [20]. The discrepancies reflect the inherent limitations of population-level and observational study designs: confounding by secular trends, changes in coding and case ascertainment, shifts in the microbial epidemiology of IE, and the difficulty of attributing a rare outcome to a specific exposure [21]. Appraised against the ROBINS-I domains [7], no study in this literature reaches low risk of bias overall, and none is likely to while randomisation is unavailable; confounding is judged serious throughout, and classification of exposure is a further serious concern in the prescription-based studies, where dispensing records serve as a proxy for actual intake and timing. Two mechanisms make this confounding difficult to address in data of this kind. Confounding by indication operates in both directions: clinicians prescribe preferentially to patients they judge highest risk, which biases towards the null, while those who receive prophylaxis are also under active cardiological follow-up, which biases away from it. And patients who attend for prophylaxis are, by construction, patients engaged with dental care—itself protective through better oral health, a pathway no published adjustment separates from the effect of the antibiotic.

One defensible reading of the contemporary observational evidence is that it has, on balance, moved in the direction of supporting a protective effect of prophylaxis in genuinely high-risk patients, and this is the shift the ESC cited in strengthening its recommendation; a reader who sets a higher evidentiary threshold, or who weights the population-level costs of antibiotic exposure more heavily, can read the same body of work as not yet warranting a change in practice [18,22]. A 2024 systematic review and meta-analysis, for example, found that prophylaxis was associated with a significantly lower risk of IE following invasive dental procedures in high-risk individuals (pooled relative risk approximately 0.41, 95% confidence interval 0.29 to 0.57) [18]; the same review found no association in those at low or unknown risk and judged the available data insufficient to support any benefit at moderate risk, which bears directly on the ESC’s Class IIb allowance for that group. Invasive dental procedures are thought to account for only a minority of IE cases, even in high-risk patients. But this evidence remains associative, susceptible to confounding, and incapable of proving causation [23]. The case for causation is partial: biological plausibility, temporality and a coherent mechanism are satisfied, but the association is of moderate strength, no dose–response relationship has been shown, and no experimental evidence exists. Consistency depends on which studies are admitted—heterogeneity within the 2024 meta-analysis was absent (I^2^ = 0%) [18], yet the studies outside its pooled estimate did not detect the effect. The confounding described above is not, however, unlimited in what it can explain: a confounder would have to be strongly associated with both prophylaxis receipt and IE to account for an effect of this size, and whether the healthy-adherer and indication effects reach that magnitude is unresolved. The constraint is symmetrical, since the null findings cannot exclude a modest benefit either. The most that can be claimed is that the data are compatible with a modest protective effect in the highest-risk group; they do not establish it, and they do not support a return to the expansive prophylaxis of earlier decades. It follows that the ESC’s elevation of prophylaxis to a Class I recommendation—a class reserved for interventions for which there is evidence and/or general agreement that a treatment is beneficial and effective—rests on general agreement rather than on evidence of the strength that designation ordinarily connotes. That is a legitimate use of the class system, which explicitly permits consensus to substitute for data, but it is easily misread by clinicians as an evidentiary claim, and this review does not endorse it as one.

## 8. Recommended Regimens and Responsible Antibiotic Use

Where prophylaxis is indicated, the recommended approach across the ESC and AHA frameworks is a single oral dose given shortly before the procedure, timed to achieve adequate serum concentrations during the period of anticipated bacteraemia; if inadvertently omitted, the dose may be given up to 2 h after the procedure, according to the AHA [4]. The standard agent in the absence of penicillin allergy is amoxicillin, with ampicillin, cefazolin, or ceftriaxone as parenteral alternatives when oral administration is not feasible. For patients with penicillin or ampicillin allergy, a cephalosporin (in the absence of a history of severe allergy to penicillins) or another suitable agent such as a macrolide or doxycycline is selected according to the specific guideline [3,4]; representative regimens are shown in Table 2. Two aspects of these recommendations merit comment. First, the regimen structure—single dose, timed to the procedure, aimed at the window of bacteraemia—is supported only by observational studies and animal models rather than by clinical outcome data [13]. No trial has tested the schedule against any alternative; the 2 g dose and 30–60 min window are validated against measured bacteraemia [10], not IE incidence. Second, the guidelines are more confident about the agent than the indication: the same documents that hedge the decision to prescribe specify drug and dose without qualification. This asymmetry is understandable, as pharmacokinetic questions are more readily investigated than epidemiological ones, but it may lead readers to infer that the underlying question is more settled than it is.

A noteworthy development in responsible antibiotic use is the removal of clindamycin from the recommended regimens by both the AHA and contemporary European guidance. Clindamycin was previously a standard alternative for penicillin-allergic patients, but it carries a disproportionate risk of adverse effects, particularly *Clostridioides difficile* colitis, relative to other options [4]. Its removal exemplifies the broader principle now embedded in the guidelines: that the harms of the antibiotic itself, both to the individual and to the wider problem of antimicrobial resistance, are weighed explicitly against the small and uncertain preventive benefit. A further practical point is that a patient already taking an antibiotic of the penicillin class for another reason should be given a prophylactic agent from a different class.

Dentists are among the highest-volume prescribers of antibiotics in ambulatory care, and much of what they prescribe as prophylaxis is unsupported by any guideline discussed here. In a US cohort of 91,438 patients covering 168,420 dental visits (2011–2015), gingival or periapical manipulation occurred at 90.7% of visits but only 20.9% of patients had a highest-risk cardiac condition; 80.9% of prophylaxis prescriptions were discordant with guidelines. Unnecessary prophylaxis was associated with prosthetic joint devices (OR 2.31; 95% CI 2.22–2.41) and implant procedures (OR 1.66; 95% CI 1.45–1.89), and was more likely when clindamycin rather than amoxicillin was prescribed (OR 1.10; 95% CI 1.05–1.15) [24]. The practical stewardship problem is therefore not the small number of correctly indicated doses but the much larger volume of prescribing that no guideline supports.

Evidence on individual harm has also become clearer. Across nearly three million prescriptions of single-dose amoxicillin prophylaxis in England, then given as 3 g, no fatal reaction was recorded, against 13 per million for clindamycin, most of them *Clostridioides difficile* infections [25,26]. The objection that a single prophylactic dose is unsafe for the individual patient is therefore not supported by these figures, and the withdrawal of clindamycin from the recommended regimens removed most of the risk on which that objection rested; the argument for restraint now rests on antimicrobial resistance rather than on individual harm. In practical terms, the greatest potential gain lies not in moving the prophylaxis threshold in either direction but in eliminating the prescribing that lies outside every guideline, and in recording each prophylaxis decision—indication, cardiac condition, procedure, agent and dose—so that it can be audited.

## 9. The Perspective of Dental Organisations

National dental bodies have, for the most part, not issued prophylaxis criteria of their own but have adopted or operationalised the cardiological guidance for the dental setting, and their statements are therefore instructive as to how the debate is translated into clinical practice. In the United States, the American Dental Association (ADA) aligns with the AHA position, advising that prophylaxis be reserved for the small subset of patients at the highest risk of an adverse outcome and that, for many patients formerly considered eligible, the risk of antibiotic adverse effects and the concern over resistance outweigh any benefit [15]. The American Academy of Pediatric Dentistry takes a similarly restrictive view, noting explicitly that the evidence for efficacy in children is limited and that prudence is required given the risks of resistance and adverse events [27]. The Canadian Dental Association likewise endorses the AHA approach, emphasising that most patients with a cardiac history do not require routine prophylaxis and that good oral health and regular dental access are the more important protective measures [28].

The most consequential dental contribution, however, has come from the United Kingdom, where the ambiguity of the NICE guidance left dentists without a workable definition of the “non-routine” situations in which prophylaxis might still be appropriate. To fill that gap, the Scottish Dental Clinical Effectiveness Programme (SDCEP) produced implementation advice, first issued in 2018 and substantially revised in a second edition in 2026, that supports dental teams in applying CG64 [29]. Its key messages are that prophylaxis is recommended or should be considered for patients with high-risk cardiac conditions, is not recommended for those at moderate risk, and that the decision for an at-risk dental procedure should be made jointly with the patient; it also identifies a specific sub-group of higher-risk patients warranting special consideration for non-routine management. Notably, the SDCEP advice aligns with the AHA and ESC on which patients should be considered for prophylaxis, even though it differs in the specific antibiotic regimens it recommends. Its practical importance was formally recognised in October 2024, when NICE amended CG64 to signpost the SDCEP advice directly, and again in December 2024, when the same recommendation and its reference to that advice were further clarified, thereby narrowing, without closing, the gap between the literal NICE position and that of the ESC and AHA [16]. The common thread across all these bodies is unanimity on the primacy of oral health and shared, documented decision-making, with the residual disagreement concerning only the narrow question of whether and when to prescribe.

### Oral Health as the Cornerstone of Prevention

One of the few points of genuine unanimity across the ESC, AHA, and NICE is that the maintenance of good oral health is more important to the prevention of oral-origin IE than any antibiotic dose before a procedure. This consensus follows directly from the bacteraemia argument: if the great majority of oral streptococcal seeding of the bloodstream arises from the cumulative effect of daily activities in the presence of dental plaque and gingival inflammation, then reducing this bacterial and inflammatory load addresses the dominant source of exposure, whereas a single prophylactic dose addresses only the small procedural fraction [10].

For the dental team, this reframes the preventive task. Rather than viewing IE prevention as a question answered solely at the moment of prescribing, the dentist’s most consequential contribution is the establishment and maintenance of periodontal and dental health in at-risk patients: effective plaque control, treatment of gingivitis and periodontitis, prompt management of dental infection, and regular review. Poor oral hygiene has itself been identified as a risk factor for IE-related bacteraemia [30]. The corollary is that high-risk cardiac patients warrant proactive, structured dental care and education, and that access to such care is a public-health priority in its own right, independent of the prophylaxis debate.

## 10. Limitations

Several limitations should be borne in mind. This is a narrative rather than a systematic review, and is therefore subject to selection bias in the choice and emphasis of sources; methodological appraisal was narrative rather than score-based, and no quantitative synthesis was undertaken. Screening and selection were performed by a single author without duplicate assessment, so selective emphasis cannot be excluded and the review does not claim the reproducibility of a systematic review. The appraisal in Section 7 is narrative: it reasons about the plausibility of unmeasured confounding rather than constituting formal quantitative bias analysis. The author has no involvement with any guideline body and declares no relevant interest, but reviews of contested topics reflect the author’s prior weighting of harms; the position adopted here—that neither the ESC nor the NICE stance is established by the evidence, and that what separates them is the evidentiary threshold each body applies rather than the data themselves—represents the author’s interpretation. The underlying evidence base is itself a fundamental limitation: the central questions (whether prophylaxis prevents IE, and in whom) have not been answered by randomised controlled trials, leaving the field reliant for the present on observational and population-level data that cannot establish causation and that are vulnerable to confounding and to changes in the epidemiology of the disease. Guidelines also evolve, and specific criteria, regimens, and wording cited here may be superseded; readers must consult the current primary documents applicable to their jurisdiction. Finally, the review centres on oral-origin, viridans-streptococcal disease amenable to dental prophylaxis and does not address in depth the prevention of healthcare-associated, staphylococcal, or device-related IE, which lies largely outside the dental remit.

## 11. Future Directions

The most valuable advances would come from study designs capable of reducing the present uncertainty without requiring an individually randomised trial, which remains impracticable. Large, linked, prospective cohort and registry studies, connecting dental, prescription, and hospital records across whole populations, offer the most realistic route to quantifying the effect of prophylaxis in genuinely high-risk patients while adjusting for confounders. Harmonisation of the international guidelines, or at least an explicit reconciliation of the ESC/AHA and NICE positions accompanied by a shared appraisal of the same evidence, would reduce the discordance that currently affects clinicians and patients. Research into the relationship between specific measures of oral health and IE incidence could strengthen the evidential basis for the prevention strategy on which all bodies already agree. Finally, work on better risk stratification, identifying which high-risk patients derive the greatest benefit, would allow prophylaxis to be targeted more precisely, maximising benefit while minimising antibiotic exposure.

## 12. Conclusions

The prevention of oral-origin infective endocarditis sits at an unusual and unresolved intersection of cardiology and dentistry. The contemporary guidelines agree on more than is sometimes appreciated: that prophylaxis, if used, belongs to the highest-risk patient; that, where a regimen is specified, the antibiotic of choice is amoxicillin and that clindamycin should no longer be used; and, above all, that the maintenance of good oral health outweighs any pre-procedural dose. Yet, on the central question of whether to offer prophylaxis at all, the strengthened Class I endorsement of the ESC and the closely aligned position of the AHA remain at odds with the long-standing NICE advice against routine use, and that discordance is real and consequential, and cannot currently be resolved on the basis of definitive evidence. This review’s argument is that the discordance reflects the limitations of the available evidence rather than an error by either body: on the evidence as it stands, the ESC’s Class I recommendation is better read as an expression of expert consensus than as a demonstrated effect, while the NICE position remains a defensible reading of the same data. Recognising this is of greater value to the clinician than attempting to determine which position is correct, because it identifies the part of the decision that the evidence can settle—which is smaller than commonly supposed—and the part that must be settled with the patient. For the dental practitioner, the constructive response is neither to disregard the issue nor to defer decisions pending a resolution that may not be forthcoming: it is to know which framework governs one’s practice, to apply it consistently, to place oral health at the centre of prevention, and to make and document decisions jointly with well-informed patients. Until stronger evidence emerges, careful, transparent, individualised practice is the most appropriate standard of care.

## Figures and Tables

**Table 1:** High-risk cardiac conditions for which antibiotic prophylaxis is considered, and points of difference between the ESC (2023) and AHA (2021) frameworks [3,4].

Cardiac Condition	ESC 2023 [3]	AHA 2021 [4]
Prosthetic valve or prosthetic valve-repair material	Highest-risk; AP recommended	Highest-risk; AP recommended
Previous infective endocarditis	Highest-risk; AP recommended	Highest-risk; AP recommended
Unrepaired cyanotic congenital heart disease	Highest-risk; AP recommended	Highest-risk; AP recommended
CHD repaired with prosthetic material (first 6 months; or lifelong if residual defect)	Highest-risk; AP recommended	Highest-risk; AP recommended
Surgical or transcatheter pulmonary valve or conduit	Covered within CHD repaired with prosthetic material; not a separate category	Explicitly listed (e.g., Melody valve, Contegra conduit)
Cardiac transplant recipient with valvulopathy	Heart transplant recipients: AP may be considered (Class IIb)	Included as highest-risk
Ventricular assist device	Highest-risk if destination therapy; AP recommended (new in 2023)	Covered within prosthetic cardiac valve or material
Moderate-risk cardiac conditions	AP may be considered individually (Class IIb)	Not recommended

AP, antibiotic prophylaxis; CHD, congenital heart disease; ESC, European Society of Cardiology; AHA, American Heart Association. Entries summarise the broad position of each body; clinicians should consult the primary documents for the authoritative and complete criteria. The table was compiled by the author from the primary documents [3,4].

**Table 2:** Representative single-dose antibiotic prophylaxis regimens for high-risk patients undergoing an invasive dental procedure (adults), based on ESC 2023 and AHA 2021 guidance [3,4].

Clinical Situation	Agent	Typical Adult Single Dose
No penicillin allergy, oral route	Amoxicillin	2 g orally, 30–60 min before procedure
Oral route not feasible	Ampicillin (or cefazolin/ceftriaxone)	2 g IV/IM (cefazolin/ceftriaxone 1 g)
Penicillin allergy, oral route	Cephalexin (if no severe penicillin allergy), azithromycin, clarithromycin, or doxycycline	Per guideline (e.g., cephalexin 2 g; azithromycin/clarithromycin 500 mg; doxycycline 100 mg)
Penicillin allergy, oral route not feasible	Cefazolin or ceftriaxone (if no severe penicillin allergy)	1 g IV/IM
Already taking a penicillin-class antibiotic	Select an agent from a different class	As above, non-penicillin option

IV, intravenous; IM, intramuscular. Doses are illustrative and simplified; exact agents, doses, and paediatric regimens differ between guidelines and jurisdictions. Clindamycin is no longer recommended. Clinicians must consult the current primary guidance applicable to their setting and the patient’s allergy history before prescribing. As with Table 1, the table was compiled by the author and necessarily simplifies some distinctions made in the original documents.

## Data Availability

No new data were created or analysed in this study. Data sharing is not applicable to this article.

## Abbreviations

AHA	American Heart Association
AP	antibiotic prophylaxis
CHD	congenital heart disease
ESC	European Society of Cardiology
IE	infective endocarditis
IM	intramuscular
IV	intravenous
NICE	National Institute for Health and Care Excellence
SDCEP	Scottish Dental Clinical Effectiveness Programme
